# Impact of a pharmacist-led educational intervention on knowledge, attitude, and practice toward the rational use of antibiotics among healthcare workers in a secondary care hospital in Punjab, Pakistan

**DOI:** 10.3389/fphar.2023.1327576

**Published:** 2024-01-29

**Authors:** Shairyar Afzal, Farman Ullah Khan, Muhammad Tahir Aqeel, Matti Ullah, Mishal Bajwa, Masoom Akhtar, Muhammad Majid

**Affiliations:** ^1^ Faculty of Pharmacy, Hamdard University, Islamabad, Pakistan; ^2^ Department of Pharmacy, District Head Quarter Hospital Jhelum, Jhelum, Pakistan; ^3^ Pharmacy Administration and Clinical Pharmacy Xian Jiaotong University, Xi’an, China; ^4^ Department of Pharmacy, Quaid-i-Azam University, Islamabad, Pakistan

**Keywords:** rational antibiotic use, ASP, antimicrobial resistance, knowledge attitude and practice, pharmacist-led educational intervention

## Abstract

**Introduction:** Growing antimicrobial resistance (AMR) and decreasing efficacy of the available antimicrobials have become a significant public health concern. The antimicrobial stewardship program (ASP) ensures the appropriate use of antimicrobials and mitigates resistance prevalence through various interventions. One of the core components of the ASP is to educate healthcare workers (HWs). Therefore, this study aims to identify the impact of a pharmacist-led educational intervention targeting knowledge, attitude, and practices regarding rational antibiotic use among healthcare professionals in a secondary care hospital in Punjab.

**Methods:** This is a single-center, questionnaire-based, pre–post interventional study conducted over a six-month time period. Data analysis was conducted using SPSS version 26.

**Results:** Regarding the pre-interventional knowledge, attitude, and practice (KAP) score of the respondents, 90.3% had a good knowledge score, 81.5% had a positive attitude, and 72.3% of HWs (excluding doctors) had a good practice score. Additionally, 74.6% of the doctors had a good practice score. After educational intervention, there was a significant improvement in the knowledge, attitude, and practice of the respondent HWs (*p*-value <0.001). Furthermore, males have higher knowledge scores compared to females in the pre- and post-intervention stages (*p*-value <0.05), and doctors differ from nurses regarding knowledge scores in both pre- and post-intervention stages.

**Conclusion:** Considering educational programs as the backbone of the ASP, it is imperative to sustain efforts in the ongoing educational programs of HWs to foster high awareness and adherence to the ASP among HWs.

## 1 Introduction

Since the discovery of antibiotics, the affirmation of a marked decrease in mortality has been indisputable. The World Health Organization (WHO) has declared inappropriate antibiotic use as a “global threat to public health” and a significant contributor toward antimicrobial resistance, causing 1.27 million deaths globally in 2019 (CDC, 2022), which could reach up to 10 million deaths per year and cost 100 trillion dollars to the global economy by 2050 (Jim O’Neill, 2016; Amaha et al., 2019; CDC, 2022; Murray et al., 2022). Thus, it is imperative to rationalize the use of antibiotics to sustain their effectiveness (Broom et al., 2015). The terminology most often used for the rational use of antibiotics within hospitals is referred to as “Antimicrobial Stewardship” (Laxminarayan et al., 2013). An essential goal of the antimicrobial stewardship program (ASP) is to ensure the appropriate usage of antimicrobials today to render them effective for those needing them in the future (Cox et al., 2017; Dyar et al., 2017).

The ASP is designed to optimize antibiotic use; education and training complement the effectiveness of ASP activities in hospitals (Apisarnthanarak et al., 2018). Educational intervention is considered a valuable tool for promoting appropriate antibiotic use (Harbarth et al., 2015; Barlam et al., 2016; Cox et al., 2017; Godman et al., 2021). In a study conducted at the National Liver Institute, Egypt, an educational program was offered to healthcare providers as an intervention to the ASP, and improvement in the knowledge, attitude, and practice of healthcare providers was observed as a result of the intervention (Tahoon et al., 2020). Various other studies have also shown significant improvements in the rational utilization of antibiotics after the educational intervention, particularly led by pharmacists (Monmaturapoj et al., 2021; Lai et al., 2022; Otieno et al., 2022; Xu et al., 2022; Almutairi et al., 2023; Lutfiyati et al., 2023). The ASP requires multidisciplinary healthcare workers to perform as a single unit, and education based on updated information is a requisite for informed decision making (Laxminarayan et al., 2013; Cosgrove et al., 2014).

Pakistan faces irrational prescribing and dispensing adversities leading to high antimicrobial resistance (AMR) (Saleem et al., 2018; Rakhshani et al., 2022). The antibiotic armamentarium has been severely compromised due to the non-judicious use of broad-spectrum antibiotics (Haseeb et al., 2022). A point prevalence survey concluded a staggering 77.6% of antibiotic use within the included hospitals of Punjab (Saleem et al., 2019), and two separate simulated client studies observed an astounding 90.5% and 96.9% of antibiotics being dispensed without prescription from pharmacies and medical stores of Pakistan (Saleem et al., 2020; Ahmad et al., 2022). Furthermore, the Pakistani pharmaceutical market is overwhelmed with “me too” generics of antibiotics, especially from the “Watch” category of WHO AWaRe classification for antibiotics, posing an extreme strain on marketing these brands and eventually increasing consumption through prescribers, ultimately taking Pakistan to the top antimicrobial consumers among developing countries (Malik and Figueras, 2019).

Pakistan’s National Action Plan (NAP) against AMR emphasizes the need for an ASP in hospital settings under the fourth strategic priority (M.o.N.H.S.R.C, 2017). However, currently, available literature portrays substantial barriers that are unaddressed, consequently leading to meager implementation (Khan et al., 2020; Saleem and Pethani, 2020; Atif et al., 2021; Mubarak et al., 2021; Chang et al., 2022). Pakistan, a resource-limited country, struggles to properly implement the ASP due to the oblivious attitude of the health professional community and the non-existence of guidelines advocating for prescribing discipline regarding the rational use of antibiotics and an effective infection control program (Atif et al., 2021). Considering AMR, a looming threat, healthcare professionals in Pakistan showed a positive attitude toward ASP implementation and offered to be obtainable to educational activities despite their lack of familiarity with the program (Hayat, Rosenthal, Gillani, et al., 2019; Hayat et al., 2020). The optimal implementation of an ASP in a hospital is a collaborative effort of all health professionals, including physicians, pharmacists, nurses, and other allied health professionals dealing with antibiotics in their roles and at different stages of a treatment cycle (Saleem et al., 2022). Pharmacists, being experts in antimicrobials and a core component of a stewardship team, can play a significant role in preventing inappropriate antimicrobial use (Goff and Rybak, 2015, p. 2015; Pollack et al., 2016). A systematic review concluded that educational intervention concomitant with other antimicrobial stewardship interventions introduced by pharmacists produced beneficial outcomes and reduced the duration of antimicrobial therapy (Monmaturapoj et al., 2021). However, limited literature is available concerning educational intervention led by pharmacists in Pakistan (Butt et al., 2019; Khan and Fang, 2021). Moreover, literary resources covering secondary care facilities concerning ASP interventions and implementation are also scarce (Rupali and Kumar, 2022). Therefore, this study aims to identify the impact of a pharmacist-led educational intervention targeting knowledge, attitude, and practices regarding rational antibiotic use among healthcare professionals in a secondary care hospital in Punjab.

## 2 Materials and methods

### 2.1 Study design and setting

A pre–post interventional cohort study was guided via a self-administered questionnaire. The educational intervention was conducted via a face-to-face lecture, assisted by an educational guide. The study was conducted in Jhelum, in Punjab province, between November 2022 and April 2023 at District Headquarter (DHQ) Hospital. It has several wards, such as the medical ward, gynecology and obstetrics ward, and pediatric ward, along with a nursery, surgical ward, cardiology ward, dialysis center, and outpatient department (OPD), offering a wide range of health services. The hospital also has an OPD pharmacy and a pharmacy in the emergency department. In general, it is a secondary care hospital with a 400-bed capacity, operating under the administrative control of the Primary & Secondary Healthcare Department, Government of Punjab.

### 2.2 Study participants

All healthcare workers (HWs) involved in prescribing, dispensing, and administering antibiotics within the confines of the health facility, including doctors, nurses, pharmacists, dispensers, and technicians/technologists, and employed by the health facility constitute the study population. A total of 256 HWs eligible for inclusion in the study were identified.

#### 2.2.1 Inclusion criteria

Eligible HWs who are acquiescent to participate fulfilled the inclusion criteria.

#### 2.2.2 Exclusion criteria

The reverse applies to exclusion criteria where HWs are non-consenting to participate and are not directly involved in prescribing, dispensing, and administering antibiotics. Moreover, HWs who are not employed in the said setup are also excluded.

### 2.3 Sampling technique and sample size

All HWs (256) fulfilling the inclusion criteria were approached throughout the study and requested to participate. More than 100 HWs could not be included because of different working shifts. Collectively, 150 HWs from all cadres agreed to participate. Among them, 20 out of 150 agreed participants who filled the pre-proforma withdrew their participation, and 6 participants were transferred to other facilities, so post-proforma could not be filled. Therefore, data collection concluded with a total of 124 participants.

### 2.4 Educational intervention

Each participant was handed the questionnaire after briefly describing the study; it was identified as pre-proforma. It took almost 10 min to fill the proforma on average. Afterward, the author provided an educational intervention via a face-to-face lecture, supported by an educational guide presented via PowerPoint. Each session lasted between 30 and 40 min. The author created the educational guide from the literature and educational material available on the CDC website (Lambrini et al., 2017; CDC, 2023). In general, the content of the educational guide comprised an introduction and classification of antibiotics, an introduction and mechanism of antibiotic resistance, the importance of rational utilization of antibiotics, and an introduction to and the importance of the ASP in healthcare facilities. Cadre-specific content included basic prescribing principles and factors to consider before prescribing for doctors, factors to consider before administering antibiotics, the mechanism of action of antibiotics, and the importance of correct dispensing and patient counseling for nurses, pharmacists, and others. It was presented by the lead author and assessed by the two experts in the field of clinical pharmacy. The author collected the data from HWs in small batches ranging from 8 to 10 participants on any day except for doctors who were being visited in groups of 2–3 participants each time. After the gap of 15 days, the author contacted each participant at their workstation and requested to fill the post-proformas.

### 2.5 Data collection tool

The questionnaire was acquired from a few similar studies and adopted as per the objectives of our study (Tegagn et al., 2017; Sarwar et al., 2018). The five-part questionnaire included a demographic section (gender, age, profession, and experience), a knowledge section (inquiring about the knowledge of HWs regarding antibiotics and AMR) including 10 questions, a section regarding familiarity with related terminologies (3 questions/terms), an attitude section (probing general attitude about antibiotics and AMR) including 6 questions, and a two-part practice section, where one part covered the generalized practices of all HWs other than doctors (nine questions) and the second part consisted of four questions, for which the doctors were meant to fill in regarding their prescribing practice. The total count of questions was 32, excluding demographics. Each question was based on a 5-point Likert scale (1 = no opinion, 2 = strongly disagree, 3 = disagree, 4 = agree, and 5 = strongly agree) except Section 3, where familiarity related to terminologies (AMR, rational antibiotic use, and ASP) was evaluated using a five-item scale [1 = not at all familiar (I have never heard of it), 2 = not familiar (I have heard the term, but I am not sure what it is), 3 = somehow familiar, 4 = familiar (I have heard the term and have some familiarity), and 5 = very familiar (engaged in practice)]. Outcome scoring was performed dichotomously as “Good” and “Poor” for the knowledge section, familiarity with terminologies and practice sections. A score of ≥70% was considered a “Good” score. For the attitude section, outcomes were dichotomized as “Positive” and “Negative,” and a score of ≥65% was considered a positive attitude.

### 2.6 Data analysis

Data analysis was performed using Statistical Package for Social Sciences (SPSS) version 26 (SPSS Inc., Chicago, IL, USA). Descriptive statistics (frequencies, percentages, mean, and standard deviation) were applied to independent variables (demographics). Data were presented in a tabulated form. A normality check for data was carried out using the Shapiro–Wilk test (where *p*-value <0.05, indicating not normally distributed data). Non-parametric statistics, including the Wilcoxon signed-rank test, was applied to continuous variables to check the differences in pre–post data, and McNemar’s test was applied to categorical variables to evaluate the differences in pre–post data. Furthermore, the independent-sample Mann–Whitney U test and Kruskal–Wallis test, followed by the Bonferroni-adjusted *post hoc* test where necessary, were applied to independent variables such as gender, age, profession, and experience. These tests were employed to check the variations within these categories concerning knowledge, attitude, and practice. A *p*-value of <0.05 was considered statistically significant, except for the *post hoc* test where Bonferroni-adjusted *p*-value was used, and all tests were two-tailed.

### 2.7 Ethical approval

Hamdard University provided ethical approval for the study to the author vide no. HU/DRA/2023/068 dated 6 February 2023. Moreover, the author was granted permission from the hospital’s medical superintendent to collect data from the participants. The objectives of the study were communicated to all the participants, and verbal consent was obtained before data collection. Participants were also ensured data confidentiality. After seeking proper consent, the questionnaire was served to the participants. Participants also had the right to withdraw from the study at any stage.

## 3 Results

### 3.1 Demographics

The sample comprised 124 HWs, primarily female, *n* = 86 (69.4%), while only *n* = 38 (30.6%) were male. Most of the HWs, *n* = 48 (38.7%), are from the 20–29 years age group category, followed by the 30–39 years age group category, *n* = 47 (37.9%). Most of the HWs, *n* = 59 (47.6%), are doctors, followed by nurses, *n* = 51 (41.1%). Pharmacists, dispensers, and others were 3.2%, 7.3%, and 0.8%, respectively. Most HWs marked experience in the category of <5 years with *n* = 49 (39.5%), followed by the >10 years of experience category with *n* = 41 (33.1%). Table 1 depicts the details of the demographic characteristics of the sample.

**TABLE 1:** Details of the demographic characteristics of the sample.

	—	n	%
Gender	Male	38	30.6
	Female	86	69.4
Age	20–29	48	38.7
	30–39	47	37.9
	40–49	21	16.9
	50–59	8	6.5
Profession	Doctor	59	47.6
	Pharmacist	4	3.2
	Nurse	51	41.1
	Dispenser	9	7.3
	Other	1	0.8
Experience (years)	<5	49	39.5
	5–10	34	27.4
	>10	41	33.1

Sample demographics (*n* = 124).

### 3.2 Difference between pre and post knowledge scores

**FIGURE 1:**
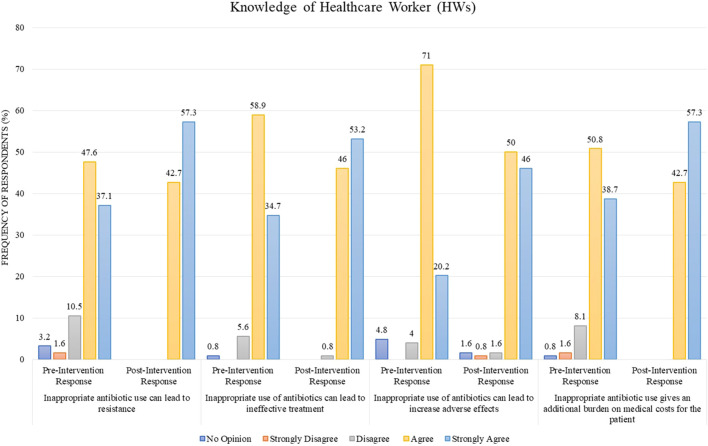
Comparison of pre- and post-intervention responses regarding the knowledge of healthcare workers.

Regarding the statement “inappropriate antibiotic use can lead to resistance,” 47.6% of HWs agreed and 37.1% strongly agreed to it in the pre-intervention stage as compared to the post-intervention stage, where 42.7% agreed and 57.3% strongly agreed (Figure 1). The statement “inappropriate use of antibiotics can lead to ineffective treatment” was agreed by 58.9% and strongly agreed by 34.7% in the pre-intervention stage, whereas the post-intervention stage showed that 46% agreed and 53.2% strongly agreed to it (Figure 1). In another statement, “inappropriate use can lead to increased adverse effects,” 71% of the respondents agreed and 20.2% strongly agreed to it in the pre-intervention stage as compared to the post-intervention stage, where 50% agreed and 46% strongly agreed (Figure 1). For the statement “inappropriate antibiotic use gives an additional burden on medical costs for the patient,” 50.8% of respondents agreed and 38.7% strongly agreed in the pre-intervention stage as opposed to the post-intervention stage, where 42.7% agreed and 57.3% strongly agreed (Figure 1).

Educational intervention regarding the rational use of antibiotics improved the percentage of “Good” knowledge among HWs. In total, 90.3% of them possessed “Good” knowledge in the pre-intervention stage compared to 100% in the post-intervention, which is a statistically significant result with a *p*-value <0.001 (Table 2). Moreover, there is a difference of mean in the pre-intervention knowledge score of HWs and post-intervention knowledge score from 78.48 ± 7.291 to 83.73 ± 6.413, which is also statistically significant with *p*-value <0.001 (Table 3).

**TABLE 2:** Total knowledge, attitude, and practice score of healthcare workers and doctors regarding the rational use of antibiotics.

	Pre-intervention		Post-intervention		*p*-value
	n	%	n	%	
Total knowledge score					
Good (≥70%)	112	90.3	124	100	<0.001*
Poor (<70%)	12	9.7	0	0	
Total attitude score					
Positive (≥65%)	101	81.5	123	99.2	<0.001*
Negative (<65%)	23	18.5	1	0.8	
Practice of healthcare workers					
Good (≥70%)	47	72.3	64	98.5	<0.001*
Poor (<70%)	18	27.7	1	1.5	
Prescribing practice of doctors					
Good (≥70%)	44	74.6	58	98.3	0.001*
Poor (<70%)	15	25.4	1	1.7	

Data were interpreted using McNemar’s test at *p* < 0.05.

**TABLE 3:** Knowledge, attitude, and practice (healthcare workers/doctors) regarding the rational use of antibiotics.

	Pre-intervention	Post-intervention	*p*-value
Knowledge	78.48 ± 7.291	83.73 ± 6.413	<0.001*
Attitude	73.63 ± 10.429	83.44 ± 9.532	<0.001*
Practice of healthcare workers	75.45 ± 7.396	81.81 ± 5.745	<0.001*
Prescribing practice of doctors	75.34 ± 12.065	86.53 ± 7.614	<0.001*

Data values are represented as mean and interpreted using the Wilcoxon signed-rank test with *p* < 0.05.

Furthermore, the independent-sample Mann–Whitney U test was applied to investigate the difference across gender categories, and a statistically significant difference (*p*-value <0.05) was found in both the pre- and post-intervention knowledge scores of the respondents. Males have higher knowledge scores than females in the pre- and post-intervention stages (mean rank = 73 and 74.93, respectively) (Table 4). The independent-sample Kruskal–Wallis test was applied to independent variables (age, profession, and experience), and a statistically significant difference (*p*-value <0.05) was found across all the independent variables for knowledge scores of the respondents in the pre-intervention stage. Except for age, a statistically significant difference (*p*-value <0.05) was found across the independent variables (profession and experience) for the knowledge scores of the respondents in the post-intervention stage (Tables 4, 5). Moreover, statistically significant variables for both pre- and post-intervention stages were subjected to the Bonferroni-adjusted *post hoc* test, which indicated that doctors were different from dispensers and nurses (*p*-value = 0.00) in the pre-intervention stage. In contrast, in the post-intervention stage, doctors differ from nurses (*p*-value = 0.00). For details, refer to Table 4.

**TABLE 4:** Comparison of the characteristics of healthcare workers and doctors with total knowledge and attitude scores before and after an intervention.

Variable	Category	Pre-intervention (knowledge)			Post-intervention (knowledge)			Pre-intervention (attitude)		Post-intervention (attitude)	
		Rank	*p*-value	Pairwise difference^a^	Rank	*p*-value	Pairwise difference^a^	Rank	*p*-value	Rank	*p*-value
Gender^b^	Male	73.00	0.03**∼**	NA	74.93	0.01**∼**	NA	54.58	0.101	58.99	0.466
	Female	57.86			57.01			66.00		64.05	
Age (years)^c^	20–29	73.27	0.004**∼**	Between 20–29 and 40–49; *p* = 0.002	70.75	0.066	No significant difference	56.63	0.125	62.59	0.868
	30–39	61.95			62.80			69.40		63.88	
	40–49	39.38			49.17			54.38		63.05	
	50–59	61.81			46.25			78.50		52.38	
Profession^c^	Doctors	80.20	<0.001**∼**	Between doctor and nurse; *p* = 0.00	74.41	<0.001**∼**	Between doctor and nurse; *p* = 0.001	64.19	0.001**∼**	62.73	0.165
	Pharmacist	92.25			100.63			105.13		96.38	
	Nurse	45.43			47.46			62.76		59.12	
	Dispenser	25.33		Between doctor and dispenser; *p* = 0.00	46.78			24.44		59.06	
	Other	104.00			116.00			121.00		117.00	
Experience (years)^c^	<5	73.71	0.009**∼**	Between <5 and >10; *p* = 0.007	70.41	0.044**∼**	No significant difference	60.90	0.864	64.13	0.134
	5–10	60.53			64.09			65.18		70.31	
	>10	50.73			51.73			62.20		54.07	

^a^ Bonferroni-adjusted *post hoc* test, *p* < 0.05.

^b^ Independent-sample Mann–Whitney U test.

^c^ Independent-sample Kruskal–Wallis test.

**TABLE 5:** Comparison of the characteristics of healthcare workers and doctors with total practice scores before and after an intervention.

Variable	Category	Pre-intervention (practice HWs)		Post-intervention (practice HWs)		Pre-intervention (practice doctors)		Post-intervention (practice doctors)	
		Rank	*p*-value	Rank	*p*-value	Rank	*p*-value	Rank	*p*-value
Gender^a^	Male	24.06	0.125	24.17	0.119	29.29	0.754	32.69	0.226
	Female	34.44		34.42		30.68		27.40	
Age (years)^b^	20–29	39.50	0.061	40.13	0.025	27.98	0.547	27.88	0.381
	30–39	33.43		33.74		34.83		29.53	
	40–49	22.60		21.40		28.17		40.42	
	50–59	40.90		40.70		26.17		34.67	
Profession^b^	Doctors	—	0.204	—	0.456	N/A		N/A	
	Pharmacist	42.00		34.50					
	Nurse	33.40		34.26					
	Dispenser	24.06		24.17					
	Other	57.00		42.00					
Experience (years)^b^	<5	35.38	0.821	35.04	0.511	29.88	0.242	29.54	0.881
	5–10	33.44		35.42		37.00		29.00	
	>10	31.52		29.98		24.96		32.04	

^a^ Independent-sample Mann–Whitney U test.

^b^ Independent-sample Kruskal–Wallis test.

### 3.3 Difference between pre and post familiarity with terminologies

**FIGURE 2:**
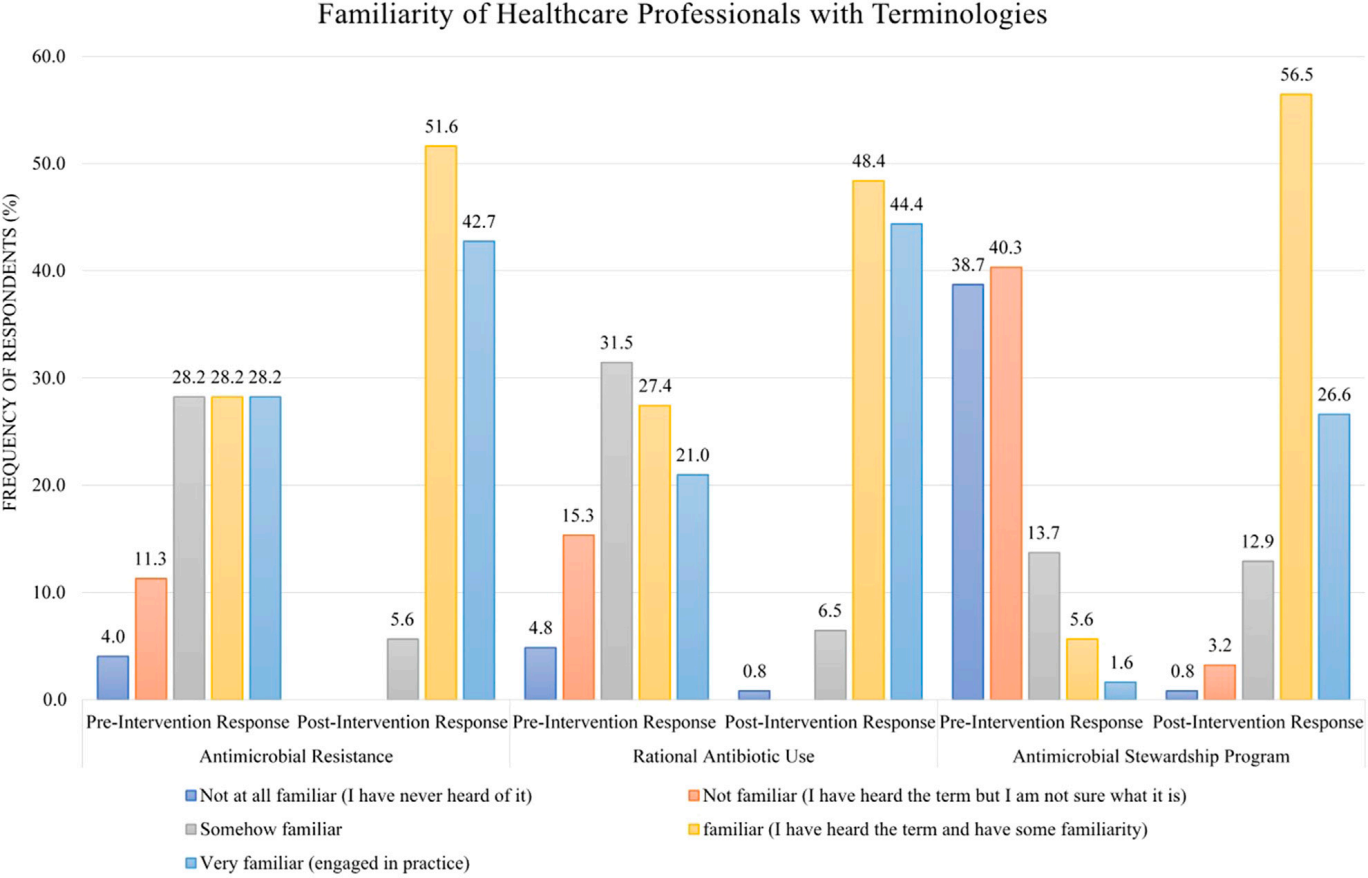
Comparison of pre- and post-intervention responses regarding the familiarity of healthcare workers with terminologies.

Regarding familiarity with terminologies, 28.2% of HWs in the pre-intervention stage were familiar with the term “antimicrobial resistance” as compared to 51.6% in the post-intervention stage (Figure 2). For familiarity with the term “rational antibiotic use,” 27.4% HWs were familiar in the pre-intervention stage as opposed to 48.4% in the post-intervention stage (Figure 2). Only few HWs (5.6%) were familiar with the term “antimicrobial stewardship program” in the pre-intervention stage as compared to 56.5% in the post-intervention stage (Figure 2).

### 3.4 Difference between pre and post attitude scores

**FIGURE 3:**
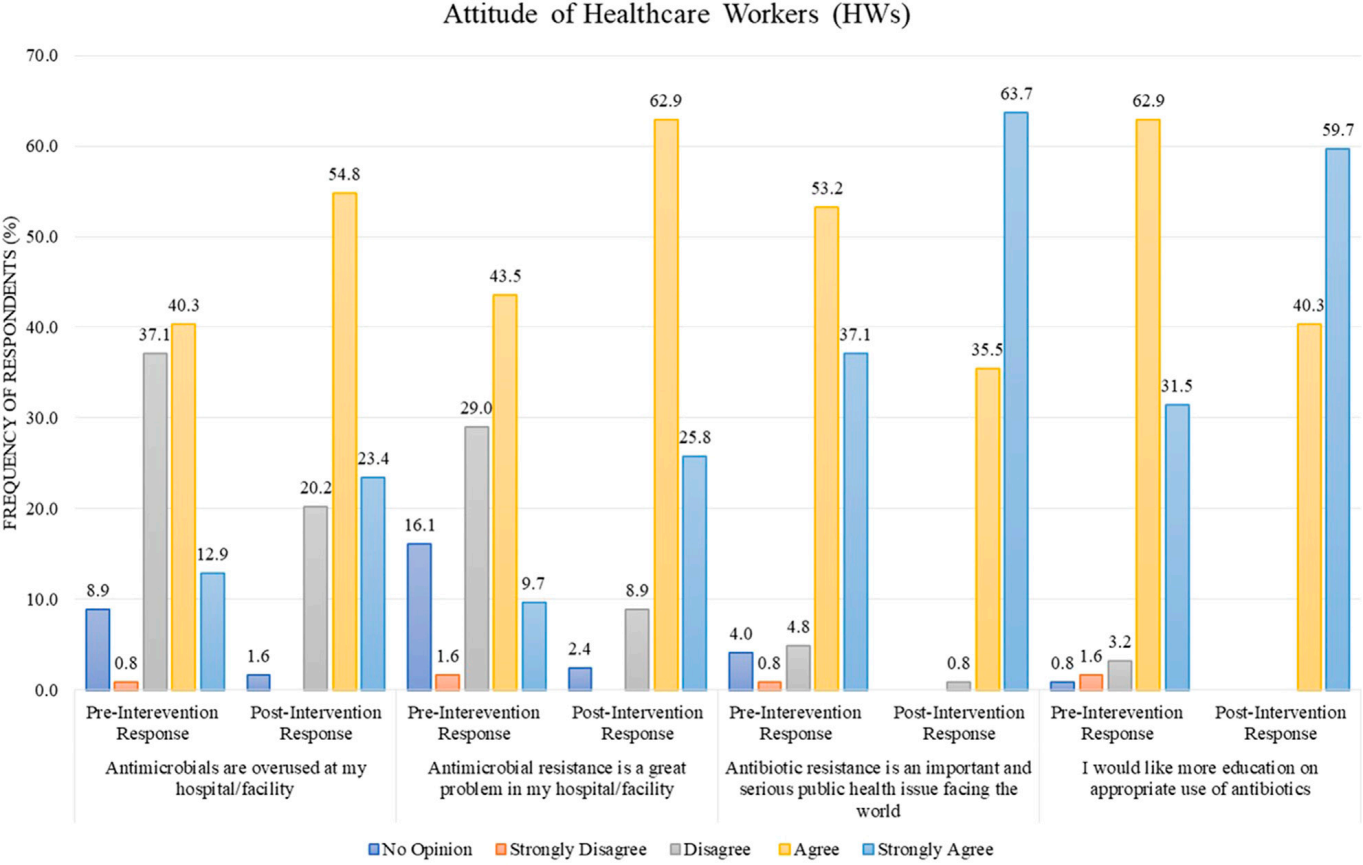
Comparison of pre- and post-intervention responses regarding the attitude of healthcare workers.

In the pre-intervention stage, the response of HWs for the statement “antimicrobials are overused at my hospital/facility” was 40.3% in agreement and 37.1% in disagreement, but after the intervention, the percentage of disagreement decreased to 20.2%, with the simultaneous increase in the percentage of agreement to 54.8% (Figure 3). For the statement “antimicrobial resistance is a great problem in my hospital/facility,” the post-intervention agreement percentage was 62.9% as compared to the pre-intervention stage, where only 43.5% agreed (Figure 3). In another statement, “antibiotic resistance is an important and serious public health issue faced worldwide,” the respondents were 53.2% in agreement before the intervention, but after the intervention, the response for this statement was converted to “strongly agree” by a percentage of 63.7% (Figure 3). The same trend was observed for the statement “I would like more education on the appropriate use of antibiotics,” with a pre-intervention agreement percentage of 62.9% as compared to the post-intervention stage where the response “strongly agree” for this statement was 59.7% (Figure 3).

The overall educational intervention improved the percentage of “Positive” attitudes in HWs regarding the rational use of antibiotics from 81.5% (pre-intervention) to 99.2% (post-intervention). This result is also statistically significant (*p*-value <0.001) (Table 2). The difference in mean between pre–post intervention scores is 73.63 ± 10.429 vs. 83.44 ± 9.532. This difference is also statistically significant, with a *p*-value <0.001 (Table 3).

### 3.5 Difference between pre and post practice scores

**FIGURE 4:**
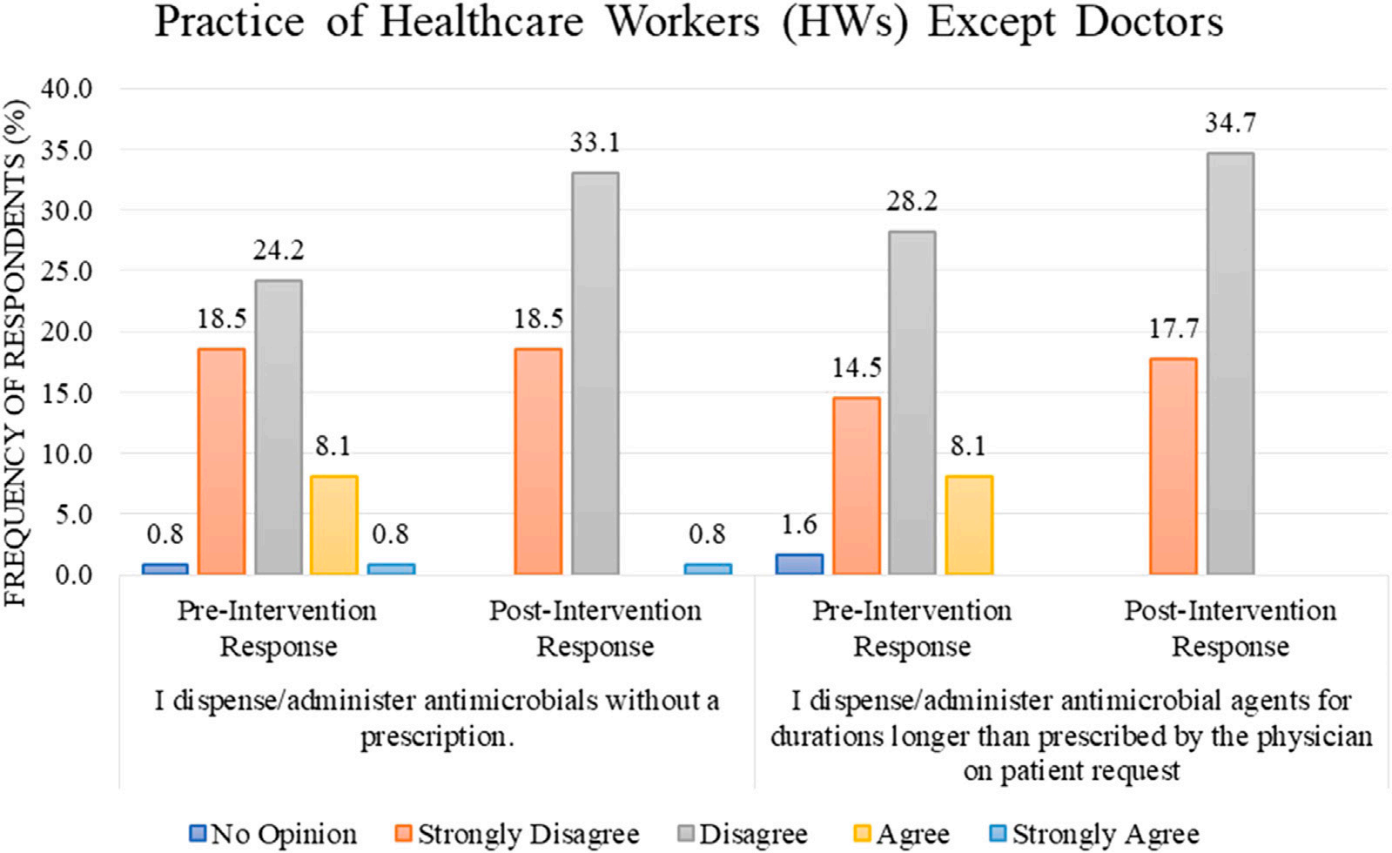
Comparison of pre- and post-intervention responses regarding the practice of healthcare workers except doctors.

Most of the HWs, other than doctors (*n* = 65, 52.4%), either disagree or strongly disagree (24.2% or 18.5%) with the statement “I dispense/administer antimicrobials without a prescription” in the pre-intervention stage as compared to the post-intervention stage where the percentage increase in the response “disagree” was observed to be 33.1%. In comparison, the response “strongly disagree” remains the same (18.5%) (Figure 4). For the statement “I dispense/administer antimicrobial agents for durations longer than prescribed by the physician on a patient’s request,” the percentage of disagree/strongly disagree response increased from 28.2%/14.5% before the intervention to 34.7%/17.7% after the intervention (Figure 4).

The percentage of HWs’ practice (other than doctors) was presented as “Good” regarding the rational use of antibiotics in response to the educational intervention; 98.5% of the respondents improved their practice after the intervention as compared to 72.3% in the pre-intervention stage, with the result being statistically significant with a *p*-value of <0.001 (Table 2). The difference in mean between pre–post intervention scores is 75.45 ± 7.396 vs. 81.81 ± 5.745. This difference is also statistically significant, with a *p*-value <0.001 (Table 3).

### 3.6 Difference between pre and post prescribing practice scores

**FIGURE 5:**
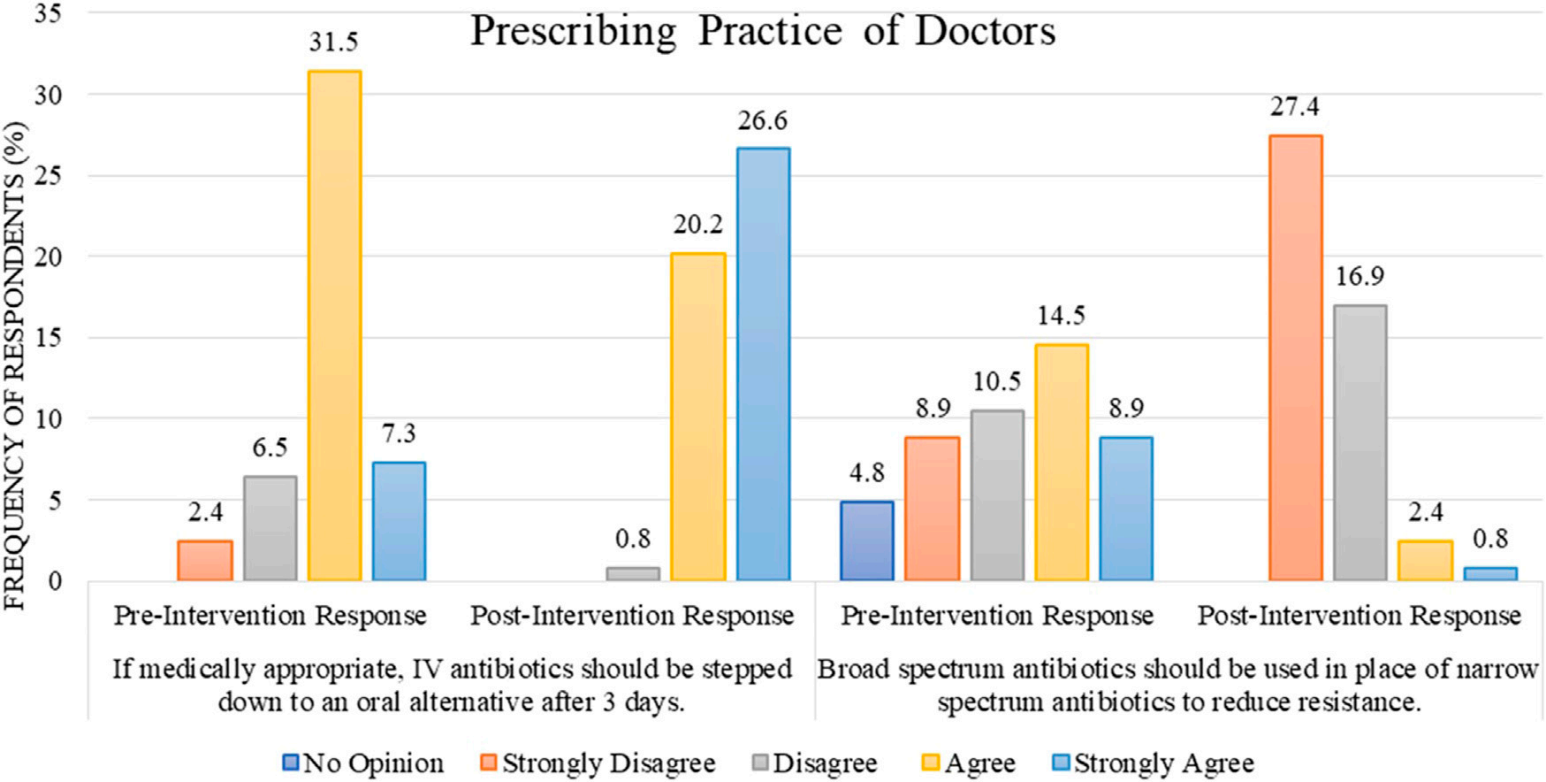
Comparison of pre- and post-intervention responses regarding the prescribing practice of doctors.

Regarding the prescribing practice of doctors (n = 59, 47.6%), most of them agreed and strongly agreed with the statement “if medically appropriate, IV antibiotics should be stepped down to an oral alternative after 3 days,” with the cumulative percentage of “agree and strongly agree” response being 38.8%/47.6% before the intervention as opposed to 46.8%/47.6% after the intervention (Figure 5).

For the statement “broad-spectrum antibiotics should be used in place of narrow-spectrum antibiotics to reduce resistance,” a range of responses were observed during the pre-intervention stage. However, after the intervention, the cumulative percentage for the responses “disagree and strongly disagree” increased to 44.3% out of 47.6% (Figure 5).

The prescribing practice of respondent doctors has been concluded as “Good” regarding the rational use of antibiotics in response to the educational intervention. During the pre-intervention, 74.6% of doctors had “Good” practice; educational intervention improved the practices, so after the intervention, 98.3% of doctors had shown “Good” practice. This result is statistically significant, with a *p*-value of 0.001 (Table 2). The difference in mean between pre–post intervention scores is 75.34 ± 12.065 vs. 86.53 ± 7.614. This difference is also statistically significant, with *p*-value <0.001 (Table 3).

## 4 Discussion

To the best of our knowledge, this is the first educational intervention study led by a pharmacist ascertaining the KAP of HWs regarding the rational use of antibiotics performed in a public hospital in Pakistan.

In general, the results of our study suggest that an educational intervention can produce a valuable improvement in KAP. Our results are similar to the previous studies, which also concluded positive outcomes after the educational intervention administered by pharmacists (Tahoon et al., 2020; Saleh, Abu Farha and Alefishat, 2021). A systematic review by Roque et al. (2014) concluded that educational intervention applied to antimicrobial use practices could produce an improved outcome.

Regarding the pre-intervention knowledge of HWs, 84.7% agreed/strongly agreed that inappropriate antibiotic use leads to resistance, like the pre-intervention response rate of other studies (Tegagn et al., 2017; Tahoon et al., 2020). In Florida, 98% of nurse practitioners agreed that inappropriate antibiotic use causes resistance (Abbo et al., 2012). A total of 93.6% of HWs agreed/strongly agreed that inappropriate antibiotic use renders treatment ineffective, similar to a study conducted in Egypt (Tahoon et al., 2020). A total of 70% of our respondents agreed that misusing antibiotics could cause harm. A Floridian study (Abbo et al., 2012) reported 96% agreement, while an Ethiopian study (Tegagn et al., 2017) reported 57.9%. This large discrepancy may be due to the developed nature of the Floridian study setting. A total of 50.8% agreed that inappropriate use increases patients’ costs (Tegagn et al., 2017).

As the educational level of different professionals are hugely varied, a statistically significant difference exists for the variable “profession” in both pre- and post-intervention stage between doctors, nurses, and dispensers regarding the knowledge of HWs. A similar trend was observed in a study conducted in South Africa (Balliram et al., 2021). For other independent variables, a statistically significant difference was found between the age category (20–29 and 40–49) and experience category (<5 and >10) in the pre-intervention stage, while in the post-intervention stage, differences across these categories were found to be non-significant. This could be because of the intervention applied and the learning effect achieved via intervention in HWs.

Before the conduction of the educational intervention, only 28.2% of the HWs were familiar with the term antimicrobial resistance, which is quite different from studies conducted in Ethiopia (Tegagn et al., 2017) and Egypt (Tahoon et al., 2020). However, another study conducted in Pakistan’s tertiary hospitals reported that physicians were highly familiar with antimicrobial resistance (Hayat, Rosenthal, Gillani, et al., 2019). This difference can be due to the inclusion of different cadres of HWs in our study. Only 5.6% of the HWs were familiar with the ASP. This finding is in line with various other studies from Nigeria (Babatola et al., 2021), Saudi Arabia (Baraka et al., 2019), Ethiopia (Tegagn et al., 2017), Egypt (Tahoon et al., 2020), and Pakistan (Hayat, Rosenthal, Zhu, et al., 2019). The possible reason could be the lack of implementation of the ASP and awareness campaigns regarding the importance of the ASP in the healthcare system. Contrary to these findings, studies from Australia (Cotta et al., 2014) and South Africa (Burger et al., 2016) showed high familiarity with the ASP. However, the familiarity rate improved to 56.5% after the intervention, indicating that the educational intervention proved beneficial in acquainting HWs with the ASP. This high awareness among developed nations is probably because of the regulatory compulsion regarding implementing the ASP in hospital settings (Australian Commission, 2011; CDC, 2019).

Like most of the studies (Tegagn et al., 2017; Baraka et al., 2019; Hayat, Rosenthal, Zhu, et al., 2019; Tahoon et al., 2020), respondents showed a positive attitude toward antimicrobial use and resistance; educational intervention only made it better. Before the intervention, almost a similar proportion of respondents agreed and disagreed (40.3% and 37.1%, respectively) regarding overusing antimicrobials at their hospital. Almost similar results were reported by other researchers as well (Abbo et al., 2012; Tegagn et al., 2017). Most of the respondents of this study agreed and strongly agreed (53.2% and 37.1%, respectively) that resistance is a serious public health issue faced worldwide, which is a similar finding to various studies reported where most respondents agreed that antimicrobial is a global problem, 95.1% (Baraka et al., 2019), 96.6% (Babatola et al., 2021), and 93.37% (Balliram et al., 2021). Only 43.5% of the respondents from our study considered antimicrobial resistance to be a problem at their hospital, which is relatively low, whereas a similar finding was reported by studies where only few respondents agreed that antimicrobial resistance was a problem at their hospital (Abera et al., 2014; Cotta et al., 2014; Hayat, Rosenthal, Zhu, et al., 2019; Balliram et al., 2021). The matter is of grave concern. Awareness campaigns regarding this falsely perceived notion of HWs and rational antibiotic use must be arranged nationally and locally. Most of the study respondents preferred education regarding correct antibiotic use, which aligns with previous studies (Abbo et al., 2012; Kalungia et al., 2019; Balliram et al., 2021).

As with the practices of HWs other than doctors, most respondents disagreed with dispensing or administering antimicrobials without prescription and for a longer duration than the physician prescribes on a patient’s request. In public healthcare institutions, it is likely that due to regular internal audits and for record-keeping sake, the practice of without-prescription dispensation or administration is avoided to the maximum extent. Instead, it is more of a community problem where dispensing antimicrobials is frequently done without a prescription. Many studies confirm this finding; a study concluded that 59.9% and 59.4% of community pharmacists dispense antimicrobials without prescription and for longer than the prescribed duration, respectively (Erku, 2016). Another study reported that 74% of pharmacists dispense antimicrobials without a prescription, mainly due to business interests (Poyongo and Sangeda, 2020). In comparison to a study where the practices of community pharmacists were deemed poor (Sarwar et al., 2018), our study concluded a good practice of HWs.

Regarding the prescribing practices of doctors, most of our study participants agreed that IV antibiotics should be stepped into oral ones, which is a similar finding reported by Tegagn et al. (2017). In contrast, the results of our study differ from those of Tegagn et al. regarding the statement, “broad-spectrum antibiotics should be used in place of narrow-spectrum antibiotics to reduce resistance.” A relatively mixed response regarding this statement was noted before the intervention, which contradicts the results reported by these studies (Abera et al., 2014; Baraka et al., 2019). However, after the intervention, most prescribers strongly disagreed with the statement, which aligns with the results reported in this study (Saleh, Abu Farha and Alefishat, 2021).

### 4.1 Challenges and future recommendations

In Punjab’s secondary care health system, there is no notified antibiotic policy or guidelines from the administrative side. Prescribing is considered the sole prerogative of physicians, with almost negligible inputs from other professional cadres, mainly pharmacists. In such circumstances, administering an educational intervention by a pharmacist is of utmost importance. Moreover, due to the lack of an established ASP in hospital settings, the custom of collaborative teamwork among doctors, nurses, and pharmacists concerning antibiotic rationalization is almost negligible, posing a serious challenge for pharmacists in devising an intervention. To conduct educational programs, the substantive support of local hospital administration and acceptance of the clinical role of pharmacists at the hospital level are imperative.

Efforts to educate HWs must continue to ensure the best patient care practices. Future studies should focus on conducting educational programs targeting the specific cadre of professionals, as per their job description and area of lacking. The efficacy of educational programs is short term (Apisarnthanarak et al., 2006; Barlam et al., 2016). A time series analysis can be beneficial in determining the efficacy of the educational intervention.

### 4.2 Strengths and limitations

This study provides the necessary confidence to the pharmacist community working in hospitals that a pharmacist-led effort regarding the implementation of the rational use of antibiotics produces beneficial outcomes. As this was a single-site study, the results obtained from this study cannot be generalized to all hospitals. Participants were enrolled via convenience sampling, so the characteristics of the clinicians who could not participate or chose not to participate are unknown. Time constraint was a limiting factor in determining sample size for data collection. Finally, the data were collected via a self-administered questionnaire, so there is a potential for response bias in the data.

This study has a few limitations. Participants were enrolled via convenience sampling, so the characteristics of the clinicians who could not participate or chose not to participate are unknown. Finally, the data were collected via a self-administered questionnaire, so there is a potential for response bias in the data.

This study has few limitations. Firstly, as this was a single-site study, the results obtained from this study cannot be generalized to all hospitals. Secondly, participants were enrolled via convenience sampling, so the characteristics of the clinicians who could not participate or chose not to participate are unknown. Thirdly, time constraint was a limiting factor in determining sample size for data collection. Finally, the data were collected via a self-administered questionnaire, so there is a potential for response bias in the data.

## 5 Conclusion

The study findings conclude that the educational intervention proved to be beneficial in improving the knowledge, attitude, and practice of healthcare workers in this hospital facility regarding rational antibiotic use. Considering educational programs as a backbone of the ASP, it is imperative to sustain efforts in ongoing educational programs of HWs to foster high awareness and adherence to the ASP among HWs.

## Data Availability

The raw data supporting the conclusion of this article will be made available by the authors, without undue reservation.
